# Association Between Health Literacy and Prehypertension in South Korean Adults: Cross-Sectional Study Using the 2023 Korea National Health and Nutrition Examination Survey

**DOI:** 10.2196/82684

**Published:** 2026-03-20

**Authors:** Jiyeon Chun, Dan Bi Kim, Suk-Yong Jang, Eun-Cheol Park

**Affiliations:** 1 Department of Public Health, Graduate School, Yonsei University, Seoul, Republic of Korea; 2 Institute of Health Services Research, Yonsei University, Seoul, Republic of Korea; 3 Department of Biohealth Industry, Policy Analysis Division, Graduate School of Transdisciplinary Health Science, Seoul, Republic of Korea; 4 Department of Health Policy & Management, Graduate School of Public Health, Yonsei University, Seoul, Republic of Korea; 5 Department of Preventive Medicine, College of Medicine, Yonsei University, Seodaemun-gu, 50-1 Yonsei-ro, Seoul, 03722, Republic of Korea, 82 2-2228-1862

**Keywords:** hypertension, prehypertension, cardiovascular diseases, CVD, public health, prevention, health literacy, awareness, social determinant of health

## Abstract

**Background:**

Hypertension represents an important global health challenge, closely linked to cardiovascular diseases and elevated premature mortality rates. Prehypertension, defined as elevated blood pressure not meeting the diagnostic criteria for hypertension, necessitates early intervention to prevent disease progression. Health literacy, defined as the capacity to comprehend and use health-related information, is a key determinant of health outcomes but has rarely been studied in the context of prehypertension prevention.

**Objective:**

This study investigated the association between health literacy and prehypertension in South Korean adults. Unlike prior research focusing on treatment adherence in diagnosed patients, this study used the most recent nationally representative data to explore how domain-specific health literacy is associated with prehypertension across various subgroups, identifying potential mechanisms for intervention.

**Methods:**

Data were obtained from the 2023 Korea National Health and Nutrition Examination Survey, a nationally representative cross-sectional study. A stratified, multistage clustered sampling design was used to invite participants. Adults aged 19 years and older (N=1873) who completed the Korean Health Literacy Index were included. Prehypertension was defined as a systolic blood pressure of 130 to 139 mm Hg or a diastolic blood pressure of 80 to 89 mm Hg. A multivariable survey-weighted logistic regression model was used to assess the associations between health literacy and prehypertension, adjusting for sociodemographic and health-related covariates.

**Results:**

Of the 1873 participants, 319 (17.0%) had prehypertension, and 1098 (58.6%) showed low health literacy. After adjustment, those with low health literacy had a 43% higher likelihood of prehypertension (odds ratio 1.43, 95% CI 1.07‐1.91) than those with high health literacy. Subgroup analyses revealed that the protective impact of health literacy is not uniform but is modulated by demographic contexts.

**Conclusions:**

The observed patterns may reflect three potential mechanisms: (1) motivation for and dependency on health information (eg, in women, middle-aged, lower education, and unemployed groups), (2) synergy between health literacy and resources (eg, in high-income, urban, married, and employer-insured groups), and (3) preventive efficacy in low-risk populations. Low health literacy was significantly associated with prehypertension, with variations across subgroups suggesting context-dependent mechanisms. Health literacy may serve as a modifiable determinant and compensatory resource for cardiovascular risk prevention, particularly in populations with limited access to health care. Targeted interventions that address domain-specific health literacy deficits are needed to reduce the prehypertension burden.

## Introduction

Hypertension is often referred to as “the silent killer” and is recognized as a leading global risk factor for cardiovascular morbidity and mortality, contributing to stroke, heart attack, heart failure, and kidney failure [1]. It is the primary individual risk factor for premature mortality, resulting in approximately 10.8 million preventable deaths worldwide each year. The global prevalence of hypertension has doubled from 650 million to 1.3 billion individuals over the past 3 decades since 1990 [1], with a similar trend observed in South Korea, where the number of patients undergoing hypertension treatment steadily increased from 6.32 million in 2018 to 7.27 million in 2022 [2].

Considering that hypertension risk increases with cumulative exposure [3], proactive care during the prehypertensive stage is crucial. Individuals in the prehypertensive stage face a higher risk of developing hypertension and cardiovascular diseases (CVDs) than those with normal blood pressure [4-6]. Recognizing this, the 2017 American College of Cardiology and the American Heart Association redefined blood pressure readings of 130 to 139 mmHg systolic or 80 to 89 mm Hg diastolic—previously termed prehypertension—as stage 1 hypertension, emphasizing the necessity for proactive intervention [7,8].

Health literacy—the capacity of individuals to obtain, comprehend, and use health information to promote and maintain optimal health—has emerged as a critical modifiable determinant in disease prevention and health-related decision-making. People with higher health literacy are less likely to experience medication issues or system process errors, and they tend to engage in healthy behaviors and actively participate in accessible health care services [9,10]. Numerous studies have indicated that low health literacy correlates with hypertension and CVD [11-14], prompting governments worldwide to acknowledge the importance of enhancing health literacy for the prevention and management of CVD and other non-communicable diseases following the 2014 United Nations General Assembly high-level meeting [15]. In the public health and clinical context, health literacy is considered a crucial concept closely linked to patient safety and is emphasized as a major health determinant [10,12,16-19].

Previous studies examining the relationship between health literacy and cardiovascular outcomes have predominantly focused on treatment adherence, behavior improvement, and patient prognosis [13,20-22]. However, the role of health literacy in the prehypertensive stage remains poorly understood, where lifestyle modification alone can prevent disease progression without pharmacological intervention. The health literacy measurement tools used in prior research were mostly designed for specific diseases or disadvantaged populations, typically focusing on the functional comprehension of health literacy related to medical terminology or hospital-centered health care systems [23]. Furthermore, using clinically measured blood pressure data alongside a comprehensive, multidimensional health literacy measure to investigate how health literacy functions across different sociodemographic contexts and which specific literacy domains are most relevant to early-stage blood pressure management would inform the design of targeted and resource-efficient health interventions.

The recent development of a comprehensive health literacy measurement tool by the Korea Disease Control and Prevention Agency, which aligns with Korea’s 5th Health Plan 2030 and reflects the Korean health environment [24], provides an opportunity to address these questions. The tool was included in the health survey section of the 2023 Korea National Health and Nutrition Examination Survey (KNHANES), where it measured the health literacy of participants alongside clinical health examinations, including blood pressure measurements, and the results were publicly released in 2025. This enables the investigation of the association between health literacy and objectively measured prehypertension in a general population context using a culturally relevant, multidimensional measurement tool.

Therefore, this study aimed to investigate the association between health literacy and prehypertension in the Korean adult population using data from the 2023 KNHANES, to identify sociodemographic and health-related subgroups most substantially affected by low health literacy in relation to prehypertension prevalence, and to examine which specific domains of health literacy deficiency are most strongly associated with the likelihood of prehypertension. This study focuses on the prehypertensive stage in a population free from chronic diseases, using clinically measured blood pressure data with a multidimensional health literacy measure.

## Methods

### Ethical Considerations

As this study is a secondary analysis of publicly available data from the KNHANES, individual informed consent was waived by the institutional review board of the KDCA (2022-11-16-R-A). No additional consent, compensation, or confidentiality agreements were required, as all data were fully anonymized prior to public release.

### Study Design

This cross-sectional study was conducted using data from the 2023 KNHANES, collected by the KDCA between January 2023 and December 2023. The study followed the STROBE (Strengthening the Reporting of Observational Studies in Epidemiology) guideline for cross-sectional studies (Checklist 1) [25]. The institutional review board of the KDCA granted approval of the study protocol (2022-11-16-R-A), and the requirement for informed consent for secondary data analyses was waived under the same approval.

### Data Source

The KNHANES employs a stratified, multistage clustered probability sampling design based on the 2021 Population and Housing Census. In 2023, 192 primary sampling units were identified nationwide, with 25 households systematically selected within each unit. Trained field staff visited selected households in advance to verify eligibility and arrange participation at mobile examination centers. The survey comprised Health Interview, Health Examination, and Nutrition surveys. Of 9825 sampled individuals, 6929 participated (response rate: 70.5%).

### Study Sample

Of the 6929 individuals invited to participate in the KNHANES, this study primarily included respondents aged 19 years and older who completed all health literacy questionnaires. Subsequently, individuals with hypertension (systolic blood pressure [SBP] ≥140 mm Hg, diastolic blood pressure [DBP] ≥90 mm Hg, or those on antihypertensive medication), diabetes (fasting blood glucose level ≥126 mg/dL, those on antidiabetes medication or insulin, medically diagnosed cases, or those with a glycated hemoglobin level ≥6.5%), or dyslipidemia (8-h fasting total cholesterol level ≥240 mg/dL, low-density lipoprotein cholesterol level ≥160 mg/dL, high-density lipoprotein cholesterol level <40 mg/dL, 12-h fasting triglyceride level ≥200 mg/dL, or those on cholesterol-lowering medication) were excluded from the study. In particular, individuals with diabetes or dyslipidemia were excluded to ensure internal validity by eliminating the confounding effects of metabolic comorbidity [26] and to minimize potential bias from disease management for these comorbidities [27,28]. Such management, including lifestyle modifications and pharmacological treatment, may independently influence blood pressure levels, thus obscuring the true, independent association between health literacy and blood pressure. Furthermore, individuals with missing values for covariates incorporated into the study model were excluded. A total of 1873 participants were included in the final study sample.

### Variables

#### Prehypertension

Prehypertension was defined as an SBP of 130 to 139 mmHg or a DBP of 80 to 89 mmHg. The KNHANES used an oscillometric blood pressure monitor (WatchBP Office; Microlife AG) for blood pressure measurements. The results and subsequent adjustments were evaluated and published by a panel of experts from the Korean Society of Hypertension and associated disciplines.

#### Health Literacy

Health literacy was measured using the Health Literacy Index [24], which was developed by adapting the integrated health literacy model proposed by Sørensen et al [29], covering the competencies of accessing, understanding, appraising, and applying health information to reflect the Korean context [24]. The measurement comprises 10 items across 4 key domains: disease prevention, which involves the ability to manage risk factors like smoking, alcohol consumption, and exercise; health care, defined as the ability to access, understand, interpret, and apply information on medical or clinical issues, such as adhering to medication and medical instructions; technology and resources, which entails the ability to access and evaluate the reliability of health information obtained through the internet or media; and health promotion, referring to the ability to make informed decisions on everyday health behaviors based on social and physical environmental determinants [24]. The 10 self-reported items were scored on a scale from 10 to 40 points. Scores of 31 or higher were categorized as “high health literacy,” whereas scores below 31 were classified as “low health literacy” in accordance with the criteria established in the research that aided in the development of the Health Literacy Index.

#### Covariates

Demographic and socioeconomic variables included sex; age (categorized in 10-year intervals from the 20s to 70s and above); highest educational attainment (below elementary school, middle school, high school, or college and higher); household income (high, medium, or low); employment status; residential area (metropolitan, city, or rural); marital status; and type of health care coverage (employer-insured, self-employed insured, or receiving medical aid). Health status and lifestyle variables included BMI (≥25 kg/m^2^ or <25 kg/m^2^); comorbidities (clinically verified elevated cholesterol level or borderline diabetes); smoking status (smoker or nonsmoker); high-risk alcohol consumption—defined as an average intake of 7 or more drinks per occasion for men and 5 or more for women, with consumption occurring more than twice per week; and physical activity—defined as walking for at least 30 minutes on 5 or more days per week or completing strength training on at least 2 days per week.

### Statistical Analysis

Participant characteristics are presented as frequency and percentage for categorical variables, and group differences were evaluated using the Rao-Scott chi-square test. Continuous variables are presented as mean and SD, and comparisons between groups were made using 2-tailed *t* tests. A multivariable survey-weighted logistic regression analysis was conducted to evaluate the relationship between health literacy and prehypertension status and to perform subgroup analyses by independent variables and health literacy domains. All statistical analyses accounted for the complex sampling design of the KNHANES by applying health examination weights provided by the KDCA. Survey-weighted analyses were performed using SAS (version 9.4; SAS Institute Inc) survey procedures (PROC SURVEYFREQ and PROC SURVEYLOGISTIC) to ensure nationally representative estimates. The same regression model was applied to assess dose-response trends (*P* for trend). A survey-weighted multinomial logistic regression model was used to evaluate the relative likelihood of differing blood pressure levels between low and high health literacy groups, adjusting for demographic, socioeconomic, and health-related covariates.

All estimates were reported as odds ratios (ORs) with corresponding 95% CIs. A *P* value of less than .05 was defined as statistically significant, and all analyses were performed using SAS. Multicollinearity was verified using variance inflation factors, confirming no significant multicollinearity among the independent variables included in the model.

## Results

**Figure 1.**
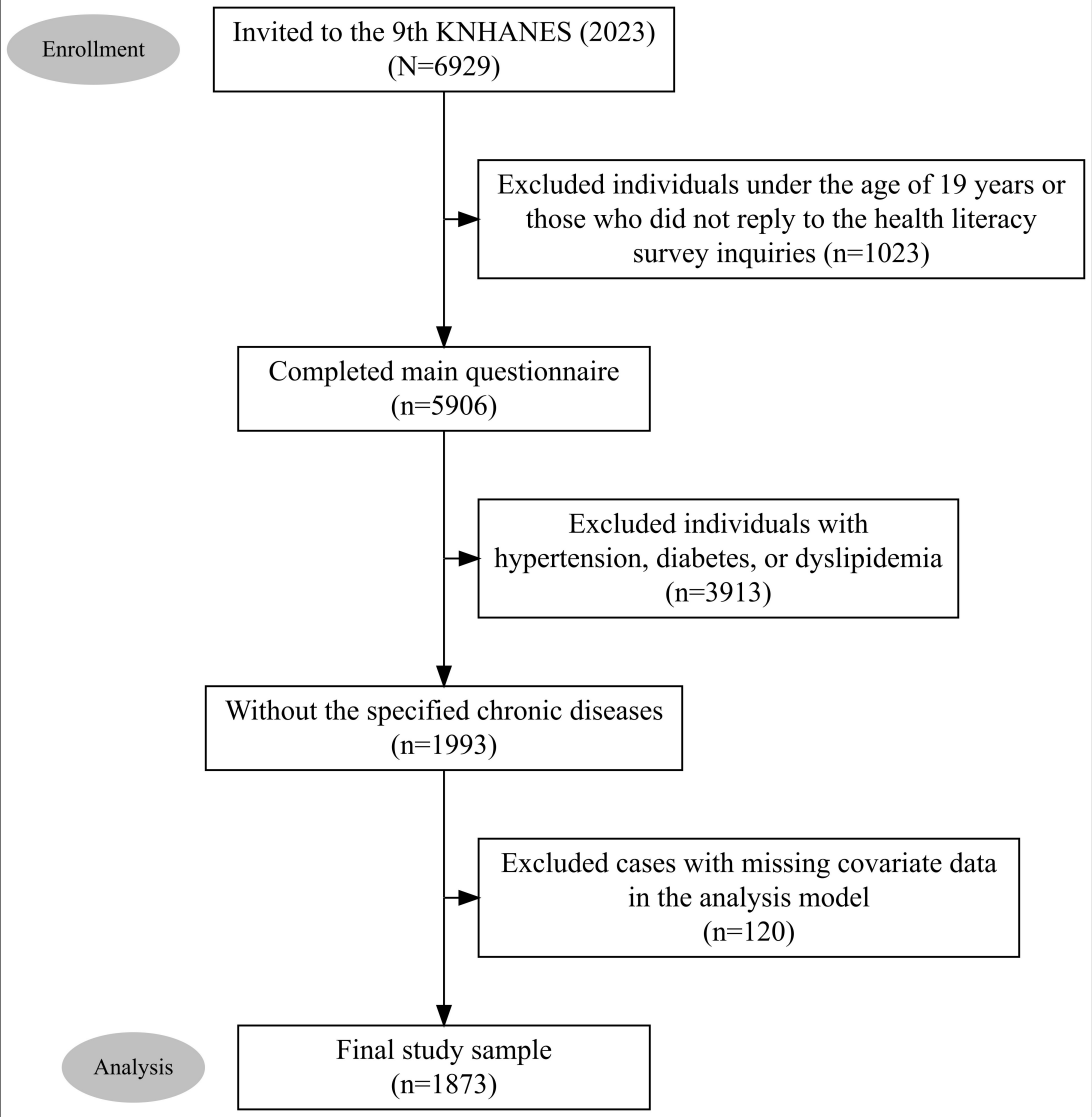
Study sample selection. Flowchart depicting the criteria for identifying the study sample for this cross-sectional study. Data were obtained from the 2023 Korea National Health and Nutrition Examination Survey (KNHANES) conducted in South Korea. The final sample consisted of 1873 participants aged 19 years or older who completed the health literacy survey; had no history of hypertension, diabetes, or dyslipidemia; and had complete covariate data.

Table 1 outlines the general characteristics of the study population, revealing that 319 (17%) participants had prehypertension among the 1873 surveyed (Figure 1). A total of 1098 (58.6%) participants were classified as having low health literacy. The distribution of health literacy differed significantly between the groups; the proportion of participants with low health literacy was notably higher in the prehypertension group than in the normal blood pressure group, as confirmed by the Rao-Scott chi-square test. Moreover, significant differences in the distribution of characteristics were observed across various factors, including sex, age, highest level of education attained, household income, marital status, type of health care coverage, BMI, presence of prechronic disease stages, smoking status, and high-risk drinking behavior.

**Table 1.** General characteristics of the study sample stratified by prehypertension status. Cross-sectional analysis of 1873 Korean adults aged 19 years or older without hypertension, diabetes, or dyslipidemia from the 2023 Korea National Health and Nutrition Examination Survey (KNHANES) conducted in South Korea. Prehypertension was defined as systolic blood pressure (SBP) of 130 to 139 mm Hg or diastolic blood pressure (DBP) of 80 to 89 mm Hg.

Variables	Total (N=1873)	Prehypertension		*P* values^a^
		Yes (n=319)	No (n=1554)	
Health literacy^b^, n (%)				<.001
Low	1098 (58.6)	228 (71.5)	870 (56)	
High	775 (41.4)	91 (28.5)	684 (44)	
Sex, n (%)				<.001
Male	659 (35.2)	158 (49.5)	501 (32.2)	
Female	1214 (64.8)	161 (50.5)	1053 (67.8)	
Age (y), n (%)				<.001
19-29	394 (21)	28 (8.8)	366 (23.6)	
30-39	374 (20)	41 (12.9)	333 (21.4)	
40-49	446 (23.8)	74 (23.2)	372 (23.9)	
50-59	275 (14.7)	56 (17.6)	219 (14.1)	
60-69	254 (13.6)	74 (23.2)	180 (11.6)	
70≤	130 (6.9)	46 (14.4)	84 (5.4)	
Educational level, n (%)				<.001
Middle school graduate or lower	199 (10.6)	63 (19.7)	136 (8.8)	
High school graduate	625 (33.4)	110 (34.5)	515 (33.1)	
College graduate or higher	1049 (56)	146 (45.8)	903 (58.1)	
Household income, n (%)				.02
High	651 (34.8)	89 (27.9)	562 (36.2)	
Middle	1024 (54.7)	179 (56.1)	845 (54.4)	
Low	198 (10.6)	51 (16)	147 (9.5)	
Work status, n (%)				.05
Employed	1249 (66.7)	196 (61.4)	1053 (67.8)	
Unemployed	624 (33.3)	123 (38.6)	501 (32.2)	
Residence, n (%)				.11
Metropolitan or city	1578 (84.2)	259 (81.2)	1319 (84.9)	
Rural	295 (15.8)	60 (18.8)	235 (15.1)	
Marital status, n (%)				.<.001
Married	1277 (68.2)	251 (78.7)	1026 (66.0)	
Single	596 (31.8)	68 (21.3)	528 (34.0)	
Health care coverage, n (%)				.009
Employee insured	536 (28.6)	111 (34.8)	425 (27.3)	
Self-employed insured	1295 (69.1)	199 (62.4)	1096 (70.5)	
Medical aid	42 (2.2)	9 (2.8)	33 (2.1)	
BMI (kg/m^2^), mean (SD)	22.8 (3.4)	24.2 (3.6)	22.6 (3.3)	<.001
Borderline dyslipidemia, n (%)				<.001
Yes	756 (40.4)	169 (53)	587 (37.8)	
No	1117 (59.6)	150 (47)	967 (62.2)	
Prediabetes, n (%)				<.001
Yes	399 (21.3)	117 (36.7)	282 (18.1)	
No	1474 (78.7)	202 (63.3)	1272 (81.9)	
Smoking status, n (%)				.048
Current smoker	234 (12.5)	50 (15.7)	184 (11.8)	
Never or former smoker	1639 (87.5)	269 (84.3)	1370 (88.2)	
High-risk drinking, n (%)				.003
Yes	183 (9.8)	46 (14.4)	137 (8.8)	
No	1690 (90.2)	273 (85.6)	1417 (91.2)	
Physical activity, n (%)				.09
Yes	1075 (57.4)	167 (52.4)	908 (58.4)	
No	798 (42.6)	152 (47.6)	646 (41.6)	

a The Rao-Scott chi-square test for categorical variables and the 2-sample *t* test for continuous variables were performed at a .05 significance level.

b Health literacy was measured using the Korean Health Literacy Index developed by Yoon et al [24]. Following the recommendations presented in the paper, health literacy scores were classified as low if <31 and as high if ≥31.

Table 2 presents the results of the multivariable survey-weighted logistic regression analysis examining the association between health literacy and prehypertension. In the crude model, individuals with low health literacy showed a significantly higher likelihood of prehypertension than those with higher health literacy. After adjusting for sociodemographic and health-related factors, this association remained statistically significant, though somewhat attenuated. The main analysis indicated that individuals with low health literacy had a 43% increased likelihood of prehypertension compared with those with higher health literacy.

**Table 2.** Association between health literacy and prehypertension in Korean adults. Cross-sectional survey-weighted logistic regression analysis of 1873 adults without chronic diseases from the 2023 Korea National Health and Nutrition Examination Survey (KNHANES) conducted in South Korea. Crude and adjusted ORs and 95% CIs are presented, with adjustments for sociodemographic and health-related covariates.

Variables	Prehypertension							
	Crude OR^a^ (95% CI)				Adjusted OR^b^ (95% CI)			
Health Literacy								
Low	1.75 (1.34-2.28)				1.43 (1.07-1.91)			
High	1.00				1.00			
Sex								
Male	2.00 (1.55-2.59)				1.86 (1.39-2.51)			
Female	1.00				1.00			
Age (y)								
19‐29	1.00				1.00			
30‐39	1.71 (0.94-3.09)				2.07 (1.12-3.83)			
40‐49	2.64 (1.54-4.52)				3.82 (2.10-6.97)			
50‐59	3.32 (1.86-5.92)				4.45 (2.28-8.70)			
60‐69	5.38 (3.13-9.25)				6.62 (3.31-13.24)			
70≤	7.07 (3.86-12.98)				6.86 (3.07-15.32)			
Educational level								
Middle school graduate or lower	3.19 (2.23-4.55)				1.26 (0.73-2.17)			
High school graduate	1.16 (0.85-1.58)				1.04 (0.74-1.47)			
College graduate or higher	1.00				1.00			
Household income								
High	1.00				1.00			
Middle	1.22 (0.84-1.77)				1.09 (0.73-1.62)			
Low	2.13 (1.27-3.57)				1.57 (0.79-3.15)			
Work status								
Employed	1.00				1.00			
Unemployed	1.33 (1.00-1.77)				1.56 (1.13-2.15)			
Residence								
Metropolitan or city	1.00				1.00			
Rural	1.31 (0.94-1.84)				0.83 (0.55-1.26)			
Marital status								
Married	1.00				1.00			
Single	0.57 (0.41-0.78)				1.36 (0.89-2.08)			
Health care coverage								
Employee insured	1.00				1.00			
Self-employed insured	0.68 (0.52-0.90)				0.87 (0.63-1.19)			
Medical aid	0.90 (0.44-1.86)				0.48 (0.19-1.26)			
BMI (kg/m^2^)	1.13 (1.09-1.17)				1.13 (1.09-1.18)			
Borderline dyslipidemia								
Yes	1.91 (1.46-2.51)				1.50 (1.12-2.00)			
No	1.00				1.00			
Prediabetes								
Yes	2.50 (1.86-3.37)				1.20 (0.87-1.67)			
No	1.00				1.00			
Smoking status								
Current smoker	1.45 (1.00-2.11)				1.16 (0.76-1.76)			
Never or former smoker	1.00				1.00			
High-risk drinking								
Yes	1.75 (1.21-2.55)				1.86 (1.21-2.87)			
No	1.00				1.00			
Physical activity								
Yes	1.00				1.00			
No	1.24 (0.96-1.60)				1.25 (0.92-1.70)			

a OR: odds ratio.

b Adjusted for sex, age, education, income, employment, residence, marriage, health care coverage, BMI, borderline dyslipidemia, prediabetes, smoking, high-risk drinking, and physical activity.

Table 3 summarizes the results of the analysis of the correlation between health literacy and prehypertension by subgroup of independent variables. The findings revealed that the association between low health literacy and prehypertension was more pronounced in certain subgroups, including women, middle-aged adults (adults in their 40s and 50s), individuals with a high school education or less, high-income households, unemployed individuals, residents of metropolitan or city areas, married individuals, individuals with employee health insurance, individuals with a BMI less than 25 kg/m^2^, nonsmokers, and non–high-risk drinkers.

**Table 3.** Subgroup analysis of the association between health literacy and prehypertension stratified by sociodemographic and health-related characteristics. Cross-sectional analysis of 1873 Korean adults without chronic diseases from the 2023 Korea National Health and Nutrition Examination Survey (KNHANES) conducted in South Korea. Adjusted odds ratios (ORs) and 95% CIs comparing low and high health literacy are presented for each subgroup.

Variables	Prehypertension: health literacy				
	High	Low, OR^a^ (95% CI)			
Sex					
Male	1.00	1.04 (0.67-1.63)			
Female	1.00	2.27 (1.48-3.47)			
Age (y)					
19‐29	1.00	1.20 (0.45-3.20)			
30‐39	1.00	0.73 (0.35-1.56)			
40‐49	1.00	2.19 (1.22-3.94)			
50‐59	1.00	3.20 (1.28-8.05)			
60‐69	1.00	1.63 (0.73-3.65)			
70≤	1.00	0.82 (0.27-2.48)			
Educational level					
High school graduate or lower	1.00	1.59 (1.01-2.53)			
College graduate or higher	1.00	1.38 (0.91-2.08)			
Household income					
High	1.00	2.21 (1.29-3.79)			
Middle	1.00	1.18 (0.79-1.75)			
Low	1.00	1.33 (0.47-3.78)			
Work status					
Employed	1.00	1.29 (0.90-1.84)			
Unemployed	1.00	1.81 (1.12-2.93)			
Residence					
Metropolitan or city	1.00	1.38 (1.02-1.87)			
Rural	1.00	1.83 (0.86-3.87)			
Marital status					
Married	1.00	1.66 (1.20-2.28)			
Single	1.00	1.10 (0.59-2.04)			
Health care coverage					
Employed insured	1.00	2.15 (1.33-3.47)			
Self-employed insured	1.00	1.23 (0.84-1.78)			
BMI (kg/m^2^)					
≥25	1.00	0.95 (0.56-1.62)			
<25	1.00	1.74 (1.17-2.60)			
Borderline dyslipidemia					
Yes	1.00	1.33 (0.86-2.05)			
No	1.00	1.49 (0.98-2.25)			
Prediabetes					
Yes	1.00	1.60 (0.92-2.78)			
No	1.00	1.34 (0.93-1.93)			
Smoking status					
Current smoker	1.00	0.82 (0.39-1.73)			
Never or former smoker	1.00	1.61 (1.18-2.20)			
High-risk drinking					
Yes	1.00	1.19 (0.54-2.62)			
No	1.00	1.49 (1.08-2.08)			
Physical activity					
Yes	1.00	1.50 (0.98-2.30)			
No	1.00	1.41 (0.94-2.12)			

a Adjusted for sex, age, education, income, employment, residence, marriage, health care coverage, BMI, borderline dyslipidemia, prediabetes, smoking, high-risk drinking, and physical activity.

Table 4 presents the results of the subgroup analyses by health literacy domain, along with the outcomes of the response trend test. Our analysis demonstrated that specific domains of health literacy, including disease prevention and health care, show a significant association with prehypertension even after adjusting for socioeconomic and health-related variables. Additionally, lower health literacy scores correlate with an increased likelihood of prehypertension, confirming statistical significance for the trend.

**Table 4.** Sensitivity analysis of health literacy domains and categorized health literacy scores for prehypertension. Cross-sectional analysis of 1873 Korean adults without chronic diseases from the 2023 Korea National Health and Nutrition Examination Survey (KNHANES) conducted in South Korea. Adjusted odds ratios (ORs) and 95% CIs are presented for health literacy domains and categorized health literacy scores. *P* value for trend is .02.

Variables	Prehypertension, OR^a^ (95% CI)			
Domains of Health Literacy Index (reference: high)				
Disease prevention	1.54 (1.10-2.17)			
Health care	1.43 (1.05-1.96)			
Technology and resources	1.38 (0.93-2.06)			
Health promotion	1.33 (0.91-1.95)			
Categorized health literacy scores				
≤23 (10th percentile)	2.10 (0.97-4.53)			
24‐32 (3rd quartile)	1.62 (0.95-2.75)			
33‐37 (90th percentile)	1.26 (0.92-2.63)			
38+	1.00			

a Adjusted for sex, age, education, income, employment, residence, marriage, health care coverage, BMI, borderline dyslipidemia, prediabetes, smoking, high-risk drinking, and physical activity.

**Figure 2.**
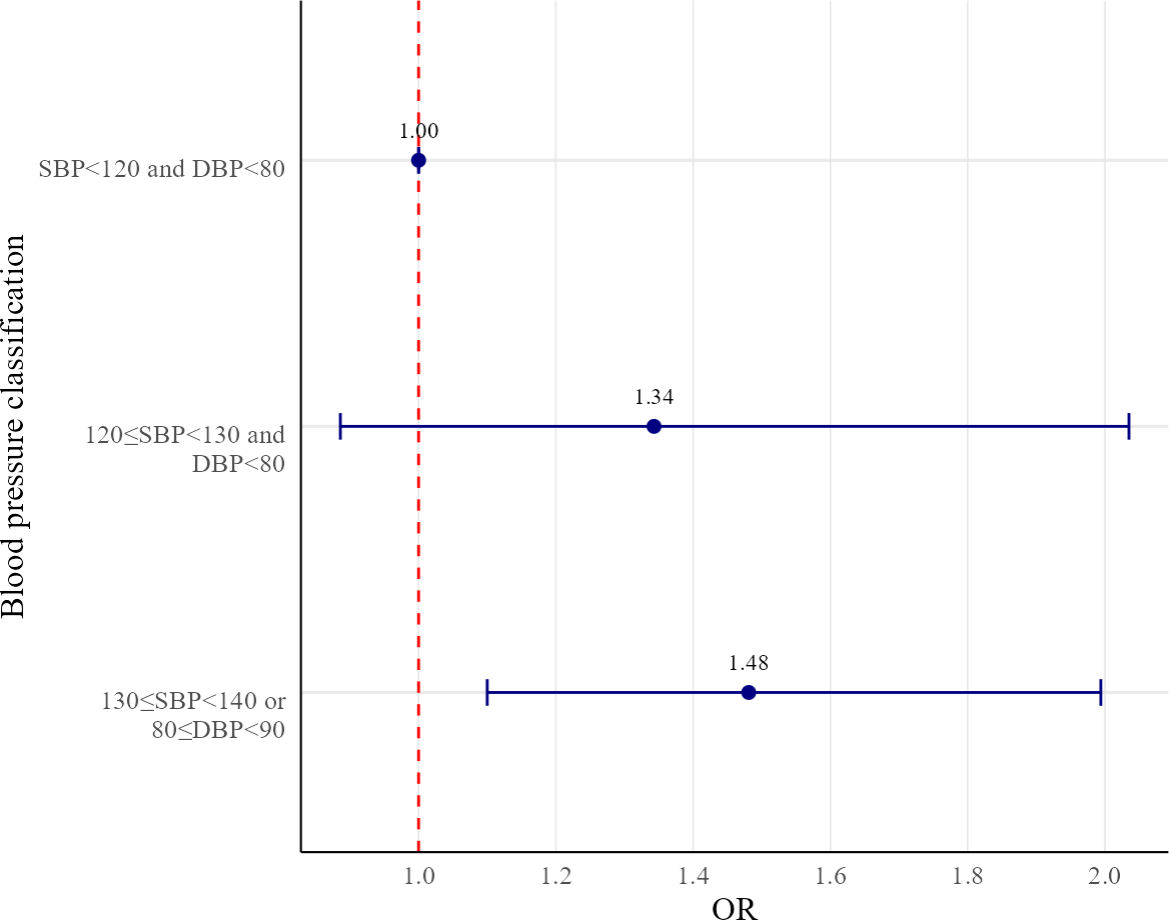
Association between health literacy and blood pressure classification in Korean adults. Forest plot derived from a cross-sectional multinomial logistic regression analysis of 1873 adults without chronic diseases from the 2023 Korea National Health and Nutrition Examination Survey (KNHANES) conducted in South Korea. Adjusted odds ratios (ORs) and 95% CIs comparing low and high health literacy are presented for blood pressure categories: normal (systolic blood pressure [SBP]<120 mm Hg and diastolic blood pressure [DBP]<80 mm Hg), caution (120≤SBP<130 mm Hg and DBP<80 mm Hg), and prehypertension (130≤SBP<140 or 80≤DBP<90 mm Hg). Models were adjusted for sociodemographic and health-related covariates.

In addition, Figure 2 illustrates the results of the multinomial survey-weighted logistic regression analysis, with the dependent variable categorized according to standard blood pressure classifications. Categorizing blood pressure measurements into three groups revealed that individuals with lower health literacy had a 48% higher likelihood of being classified in the prehypertension stage than those with normal blood pressure. Specifically, the ORs were as follows: normal group (SBP<120 mm Hg and DBP<80 mm Hg; OR 1.00, reference group), caution group (120 mm Hg ≤SBP<130 mm Hg and DBP<80 mm Hg; OR 1.34, 95% CI 0.89‐2.04), and prehypertension group (130 mm Hg ≤SBP<140 mmHg and 80 mm Hg ≤DBP<90 mm Hg; OR 1.48, 95% CI 1.10‐1.99).

## Discussion

### Principal Findings

This cross-sectional, population-based study revealed a significant association between low health literacy and prehypertension, thereby confirming a negative correlation between health literacy and blood pressure levels. This association remained statistically significant even after adjusting for sociodemographic and health-related factors, indicating a tendency for the strength of the relationship to increase as health literacy decreased and blood pressure increased. The relationship was particularly significant among specific subgroups, including women, middle-aged adults, individuals with a high school education or less, those in high-income groups, unemployed individuals, urban residents, persons with a BMI of 25 kg/m^2^ or lower, nonsmokers, and individuals who were not high-risk drinkers. This study provides evidence for preventive intervention strategies in the initial phase of blood pressure elevation.

Previous studies have investigated the association between health literacy and CVD, including hypertension, from multiple perspectives. Studies involving patients with hypertension have consistently shown that low health literacy is associated with greater difficulty in controlling blood pressure, often resulting in inadequate self-management behaviors, such as poor medication adherence and unhealthy lifestyle habits [11,12,30-34]. Furthermore, studies focused on the general adult population have indicated that higher health literacy correlates with improved management and prevention of cardiovascular risk factors including blood pressure [13,14,35]. Additionally, objective CVD risk indicators, such as the Framingham risk score, have been shown to vary based on health literacy levels [14]. Health literacy has also been recognized as playing a crucial role in medication adherence, self-management, and effective communication with health care providers [31,33,36,37].

Domestic studies using population-based data, including health literacy measurement tools such as the Korean Health Panel, have so far been limited by the absence of clinical examinations during data collection [38,39]. This has resulted in discrepancies from actual diagnostic criteria, as disease prevalence information was predominantly gathered through surveys. This study classified blood pressure based on clinical diagnostic criteria, using clinical examination data from the KNHANES, and focused on a general population free from major chronic diseases.

The main analysis results indicated a significant association between low health literacy and prehypertension. Given the increased likelihood of developing hypertension during the prehypertensive stage, these findings were consistent with those of most previous studies investigating the correlation between health literacy and blood pressure [11-14,36]. In particular, Bonaccorsi and Modesti [36] reported that health literacy and lifestyle contributed to 21.7% of the variance in hypertension. They suggested that improving health literacy could be an effective strategy for managing hypertension through patient empowerment. The American College of Cardiology and the American Heart Association noted that low health literacy is associated with reduced medication adherence and higher readmission rates, underscoring its importance in the primary and secondary prevention of CVDs [13].

The association between health literacy and prehypertension was particularly significant in certain subgroups, suggesting that the protective impact of health literacy is not uniform but may be modulated by demographic and behavioral contexts. These observed patterns can be explained by three key mechanisms: (1) the motivation for and dependency on health information (eg, in women, middle-aged individuals, individuals with lower education, and unemployed individuals), (2) the synergy between health literacy and resources (eg, in high-income, urban, married, and employer-insured groups), and (3) the preventive efficacy of health literacy in low-risk populations.

First, the associations observed among women, middle-aged adults, individuals with lower educational levels, and unemployed individuals demonstrate how behavioral motivation and the relative importance of health literacy differ across groups. Regarding women and middle-aged people, the stronger association likely indicates a heightened motivation for health management. Studies have shown that women are more likely to use health services and comply with lifestyle advice than men [40-45], and middle-aged people often experience increased health concerns that drive active engagement [45-47]. Thus, in these groups, higher health literacy is more effectively converted into tangible actions. In contrast, for individuals with lower educational levels and those who are unemployed, the finding may be explained by a resource substitution effect. Unlike those with higher education or stable employment who benefit from various cognitive resources or workplace structures, vulnerable groups often lack these buffers [48,49]. Consequently, they must rely more heavily on their particular health literacy competencies as a primary resource for navigating disease prevention, making literacy a more critical determinant of health outcomes in these groups.

Second, the notable associations among individuals with a high household income, metropolitan or city residents, married individuals, and those with employee health insurance contrast with findings from prior studies that often highlighted correlations in socially disadvantaged groups [50,51]. This finding suggests a synergy between health literacy and available resources. High household income and urban living offer the infrastructure to engage with health information [52,53], while stable employment with health insurance ensures institutional access to regular health screenings and economic stability [49]. Furthermore, spousal support provides crucial social reinforcement [54,55]. In resource-rich environments, health literacy can be effectively enacted, amplifying its protective effect against prehypertension. It is noteworthy that although the point estimate for rural residents was considerable, it did not reach statistical significance, likely due to the smaller sample size and wider CIs.

Finally, the significant correlation observed among individuals with healthier lifestyles (eg, nonobese individuals [BMI <25 kg/m^2^], nonsmokers, and moderate or nondrinkers) underscores the preventive potency of health literacy. In populations with a lower burden of risk factors, health literacy may serve as a critical primary defense, enabling early self-monitoring and the maintenance of good health behaviors before physiological dysregulation becomes irreversible [56]. This implies that health literacy is especially effective in the early stages of prevention, functioning as a key determinant for sustaining normal blood pressure in healthy individuals.

Within the domains of health literacy, disease prevention and health care–related literacy demonstrated notable associations with prehypertension. Drawing on the conceptual framework and measurement items established in our study [24,29], several behavioral mechanisms explain these associations. First, regarding disease prevention literacy, individuals with higher literacy are more likely to make informed decisions to adopt healthy behaviors than their counterparts [24]. They can better comprehend and apply recommendations such as quitting smoking, reducing salt intake, and exercising regularly, which are critical for maintaining normal blood pressure [57,58]. Conversely, those with lower literacy may struggle to grasp the risks associated with prehypertension and the necessity of these lifestyle modifications [59]. Second, regarding health care literacy, higher literacy correlates with improved adherence to medical advice and effective communication with health care providers [24]. This competence facilitates consistent blood pressure monitoring, symptom recognition, and timely medical care seeking [31-33]. Individuals with lower health literacy often encounter difficulties in performing these self-management activities, leading to poor blood pressure control. To address these deficits, multilevel intervention strategies are needed. For disease prevention, educational programs should prioritize practical methods (eg, interpreting nutritional labels) over passive information delivery [60]. For health care literacy, implementing “plain language” initiatives and training providers in the “teach-back” method are necessary to ensure that patients correctly understand and adhere to management protocols [61-64].

Our study, along with prior studies, highlights health literacy as a significant determinant of hypertension. Furthermore, enhancing health literacy via strengthened communication with health care providers and education on disease prevention may be effective in improving clinical outcomes. Therefore, enhancing health literacy is crucial for empowering patients and for the prevention and management of hypertension. Improving individual health literacy involves providing tailored educational materials and intervention programs for the general population and vulnerable groups, as well as implementing health literacy-friendly frameworks within health care institutions [13,36]. Additionally, a cohort study by Halladay et al [57] suggested that the implementation of stepwise intervention tailored to varying health literacy levels can help bridge literacy disparities and improve long-term blood pressure control, even among individuals with differing degrees of health literacy.

Building on these general principles, our findings suggest specific strategies for implementation that align with the identified mechanisms of motivation, the synergy effect with health care resources, and preventive efficacy. First, for public health authorities, given the strong association between disease prevention literacy and prehypertension revealed in our study, integrating health literacy screening with the Health Literacy Index into National Health Screening Programs is recommended. This approach capitalizes on the preventive potency of literacy observed in our low-risk subgroups. Identifying literacy deficits allows for proactive interventions to address lifestyle risks crucial for preventing the progression to hypertension. The scale is highly feasible owing to its simplicity and established cutoff points. We further propose that screening should ideally be accompanied by a follow-up system, such as easy-to-understand reports or referrals for targeted education, where results prompt practical interventions to support lifestyle modification. Second, for clinicians, addressing health care literacy is essential for the effective management of prehypertension. Implementing screening is achievable through a “waiting room screening” approach, allowing patients to self-administer the assessment prior to their visit. This provides immediate information to adapt communication, a critical step for creating synergy with health care resources for vulnerable patients who rely heavily on provider guidance due to limited external resources. By tailoring explanations to the patient’s literacy level, clinicians can bridge the motivation gap and ensure patients accurately understand prehypertension management guidelines. Moreover, the future development of a validated short-form version would likely expedite its adoption in busy clinical settings.

This study has some limitations that should be acknowledged. The cross-sectional study design precludes causal inference regarding the relationship between low health literacy and prehypertension, as temporal sequencing cannot be determined. However, to minimize bias, individuals with chronic diseases were excluded, and adjustments were made for potential confounders to clarify the association under comparable conditions. Nevertheless, while this exclusion enhanced internal validity for prehypertension assessment, it may underestimate the broader impact of health literacy on cardiovascular health across varied groups. Even after adjusting for numerous sociodemographic and health-related confounders, the potential residual confounding from unmeasured or unadjusted variables remains a possibility. Specifically, complex factors such as psychosocial characteristics, dietary patterns, and health care access were not included in the model. Since these variables may function as potential mediators in the pathway between health literacy and blood pressure, adjusting for them could risk overadjustment and obscure the direct association of interest. Additionally, caution is needed when generalizing the findings. As this study was conducted in South Korea, the results may not fully capture the dynamics in settings with distinct social, cultural, and healthcare systems. Furthermore, the exclusion of participants with diabetes and dyslipidemia resulted in a healthier analytic sample; thus, our findings may not fully apply to broader populations with metabolic comorbidities. Finally, while the study used a context-specific Health Literacy Index, self-reported measures are inherently susceptible to response bias.

Despite these limitations, this study has several methodological strengths. It used a health literacy assessment tool specifically developed for the Korean population and health care context, thereby increasing its contextual relevance and strengthening the study’s internal validity. The use of clinically measured blood pressure data, rather than self-reported measures, enhanced the accuracy and reliability of the results. Thus, this methodological approach yielded a more robust dataset, facilitating a clearer interpretation of the relationship between health literacy and objectively measured health outcomes. Furthermore, the use of weighted survey data ensured a representative sample reflective of the South Korean population, thereby enhancing the external validity of the findings.

### Conclusions

The findings of this study demonstrate a significant association between limited health literacy and the presence of prehypertension, underscoring the importance of domain-specific health literacy in the early stages of hypertension development. These findings highlight the role of health literacy as a compensatory resource and modifiable determinant for populations with limited health care access. Targeted interventions that address specific health literacy deficits, particularly in disease prevention and health care navigation, may offer a practical pathway to reduce the prehypertension burden and cardiovascular risk disparities.

To advance this field, future research should use longitudinal or prospective cohort designs to establish causal relationships between health literacy and the progression from prehypertension to hypertension. Incorporating behavioral and lifestyle factors, including diet quality, stress levels, and health care use patterns, would provide a more thorough understanding of the mechanisms linking health literacy to blood pressure management. Additionally, conducting qualitative evaluations or mixed methods strategies could help in understanding the complicated aspects of health literacy, such as communication barriers and cultural perceptions of disease prevention. Comparative analysis across diverse groups or regions would improve external validity and generalizability. Finally, evaluating the effectiveness of targeted interventions that enhance particular domains of health literacy would yield practical insights for public health and clinical practice.

## Supplementary material

10.2196/82684 Checklist 1 STROBE checklist.

## Abbreviations

CVD: cardiovascular disease
DBP: diastolic blood pressure
KDCA: Korea Disease Control and Prevention Agency
KNHANES: Korea National Health and Nutrition Examination Survey
OR: odds ratio
SBP: systolic blood pressure
STROBE: Strengthening the Reporting of Observational Studies in Epidemiology
